# Nitrogen Use Efficiency of Coffee at the Vegetative Stage as Influenced by Fertilizer Application Method

**DOI:** 10.3389/fpls.2017.00223

**Published:** 2017-03-02

**Authors:** Alveiro Salamanca-Jimenez, Timothy A. Doane, William R. Horwath

**Affiliations:** ^1^Land Air and Water Resources Department, University of California, DavisDavis, CA, USA; ^2^National Center for Coffee Research, CenicaféManizales, Colombia

**Keywords:** fertilization, labeled urea, surface applied, incorporated, *Coffea arabica*

## Abstract

Nitrogen (N) is the most limiting nutrient for coffee production in Colombia. An adequate supply is especially important during the vegetative period of growth, since any deficiency during this short period is known to have lasting effects on subsequent coffee bean production. Urea fertilizer is commonly applied on the soil surface since steep slopes hamper incorporation into soil, a practice which increases the risk of N volatilization. Little information is available on N recovery during early growth stages under different fertilizer application practices. The aim of this study was therefore to provide a comparison of ^15^N uptake during the early vegetative growth stage under surface-applied and incorporation practices at two contrasting locations. The highest proportion of plant N derived from fertilizer (Ndff) occurred 60 days following application at the site with greater precipitation and soil organic matter, where surface application also increased the Ndff in roots and stems after 120 days. Although fertilizer N supplied approximately 20–29% of total plant N after 4 months, this fertilizer-derived N corresponded on average to only 5% of the total application, indicating that very little fertilizer (relative to how much is applied) reaches plants during this time. Apart from the difference in Ndff observed at the wetter site, there was no effect of application method on dry weight and macronutrient content in different plant components, root to shoot ratio, and leaf ^13^C content. However, site effects were registered for most of these measurements, with the exception of total nutrient uptake. Similarly to Ndff trends, lower root/shoot ratio and higher concentrations of N, K, and Mg in aboveground biomass were found in the site with higher rainfall and soil organic matter, likely resulting from higher soil water and N availability. These findings provide new information useful as a direction for further research looking toward increasing NUE during the vegetative stage in Colombian coffee crops.

## Introduction

Coffee (*Coffea arabica* L.) is one of the most valuable perennial crops grown globally. Under a conventional system of coffee production the crop cycle is comprised of four growth stages: (1) the germination stage takes 2 months, (2) the nursery stage lasts 6 months, (3) following transplanting to the field, the vegetative stage lasts 12–15 months, and (4) the reproductive stage continues for 4 or 5 years until productivity declines (Arcila, 2007) and another crop cycle must be initiated by stem trimming or total renewal.

Most fertilization studies have been conducted during the reproductive stage but little has been reported for the vegetative stage, which is equally important since early vigor influences subsequent productivity (Salazar, 1996).

The most indispensable nutrient for coffee production is nitrogen (N), and yield losses of up to 60% occur when no N fertilizer is applied during the reproductive stage (Sadeghian, 2008). Support for coffee crops during the vegetative stage therefore primarily consists of adequate levels of N, followed in certain cases by phosphorus, potassium, calcium or organic matter (Sadeghian, 2008). To ensure N requirements are adequately satisfied, large amounts of N are typically applied during the vegetative stage, which often exceed the maximum dose required by the plant.

In Colombia, recommendations call for increasing doses of N at 2, 6, 10, 14, and 18 months after transplanting, depending on soil organic matter content and water availability. Urea is the most common source due to its high N content (46%) and low price per N unit, although when the soil P content is low, diammonium phosphate (DAP) (18% N) is also applied. The total amount of N applied during this stage ranges from 100 to 125 grams of urea per plant, which is equivalent to rates of up to 600 kg (first year) or 650 kg (second year) of urea per hectare for plantations with densities of 10,000 plants ha^−1^ (Sadeghian, 2008).

Although it has been shown by Fenilli et al. (2007) that coffee plants treated with annual N rates from 280 to 350 kg.ha^−1^ may reabsorb up to 43% of a fertilizer N application as gaseous ammonia (NH_3_), at least 30% of the 360 kg.ha^−1^ of the N of surface-applied urea can be lost by volatilization (Leal et al., 2010). Furthermore, from 30 to 55% of an application of 250 kg.ha^−1^ of urea-N was leached as ${\text{NO}}_{3}^{-}$ (Cannavo et al., 2013). Such significant net loss of N causes important environmental concerns such as water contamination, soil acidification, greenhouse gas emissions or N volatilization, as well as economic impacts that threaten sustainability and the coffee farmer's livelihood.

Cannavo et al. (2013) also state that when previous fertilization is considered in the N balance, most coffee cropping systems are already N saturated, leading to N use efficiencies lower than 25% during the reproductive stage.

To date, no studies for the vegetative stage have been reported, but due to the lower crop N requirement during this period, N use efficiency (NUE) is likely much lower than during the reproductive stage. Combined with the high costs of N fertilizers in recent years, this emphasizes the need to optimize fertilization practices to reduce N losses and increase NUE without compromising crop yield. Knowing the fate of fertilizer N will help balance plant requirements with yield and environmental concerns (Fritschi et al., 2004), refining current management practices to maximize NUE by minimizing losses to the environment (Nielsen, 2006).

The two most commonly used methods to measure fertilizer NUE are the *difference method*, which uses the difference in N uptake between fertilized plants and non-fertilized plants, and the *isotopic method*, which directly measures the amount of N derived from the applied fertilizer and allows estimates of residual effects for both soil and plants in subsequent crop cycles (Hofman and van Cleemput, 2004).

As one of the first studies using stable isotopes under Colombian field conditions, we aimed to quantify growth, N uptake, and NUE during the vegetative stage as influenced by two fertilizer application methods in two contrasting experimental localities, where the highest and the lowest yield responses to N fertilization have been previously observed. Due to this difference in response, we hypothesized that NUE would be different between application methods as well as between sites. This study aimed to contribute to a better understanding of N processes in the soil—plant system in Colombia, in order to generate future recommendations leading to more efficient N use in the long term from both economic and agronomic standpoints.

## Materials and methods

This experiment was carried out from August to December 2012 under field conditions at two experimental stations of the National Coffee Research Center—Cenicafe known as Naranjal and Paraguaicito. These sites were chosen based on the coffee yield response to N fertilizers described by Sadeghian (2008). Among 28 sites studied, the lowest response was registered at Naranjal, while the highest one was detected at Paraguaicito, due mainly to the difference in organic matter content, which is the main soil property used in formulating N recommendations to coffee growers. Organic matter and other soil properties for both sites are presented in Table 1.

**Table 1:** **Soil properties for the experimental sites**.

**Site**	**pH water**	**Sand**	**Silt**	**Clay**	**OM**	**CEC**	**K**	**Ca**	**Mg**	**Al**	**P**
		**— — — — — — — — — — % — — — — — — — —**				**— — — — — — — — — — cmol_**+**_ kg^**−1**^ — — — — — — — —**					**mg kg^−1^**
Naranjal	4.8	49	32	19	11.3	23	0.11	0.6	0.2	2.4	2
Paraguaicito	5.2	54	27	19	7.1	13	0.34	1.7	0.3	1.7	12

*OM, Organic matter; CEC, Cationic exchange capacity*.

*Adapted from Leal et al. (2010) and Arias et al. (2009)*.

**Figure 1:**
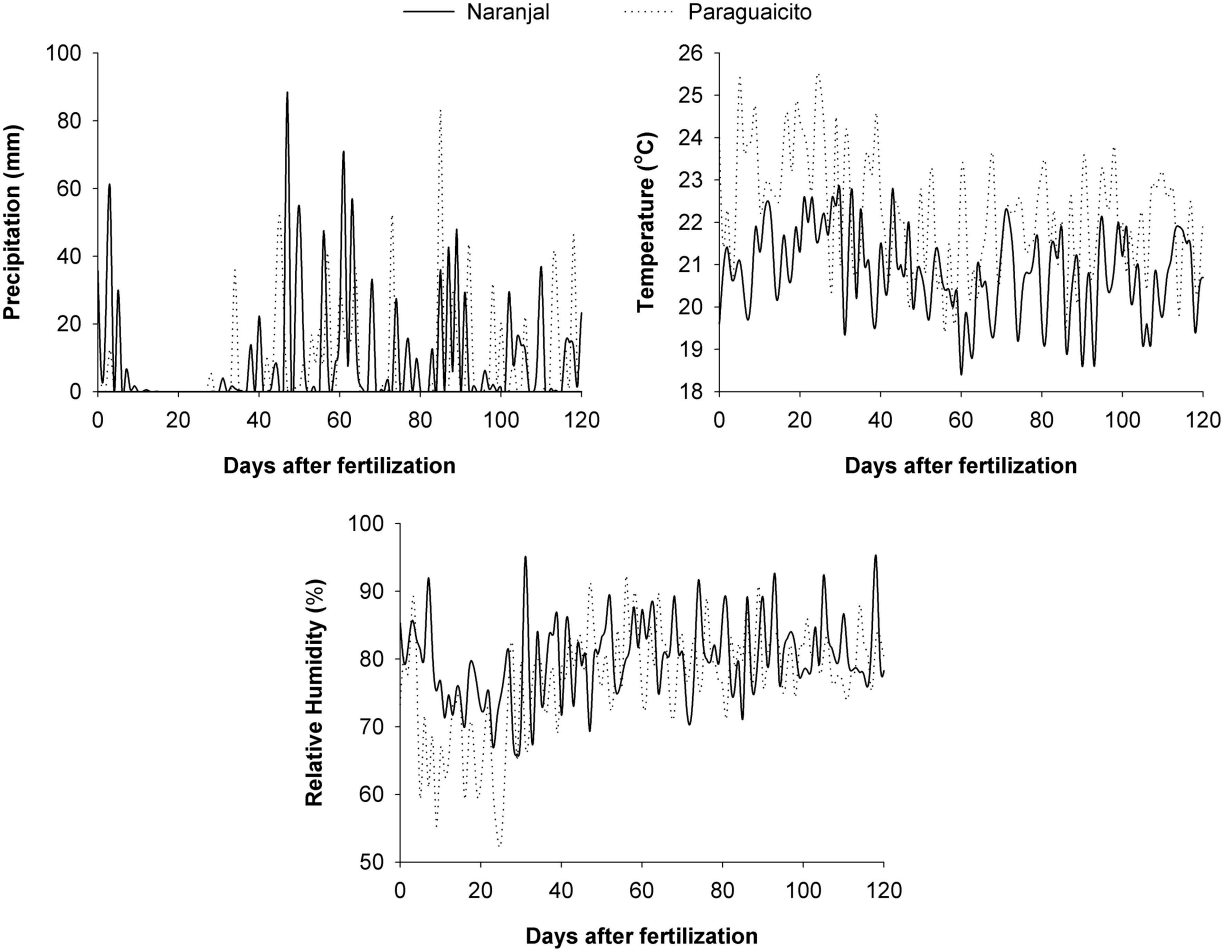
**Climatic conditions registered on a daily basis at Naranjal (solid line) and Paraguaicito (dotted line) during the evaluation period**.

Data for daily precipitation, temperature, and relative humidity were taken from a meteorological station located at each site and used to estimate the prevailing climatic conditions. During the evaluation period, a mean temperature of 21 and 22°C, an average relative humidity of 80 and 77%, and a total precipitation of 1,125 and 875 mm were registered for Naranjal and Paraguaicito, respectively. More detailed data for these variables are presented on a daily basis in Figure 1.

One coffee plantation established with no shade and a high density (10,000 plants.ha^−1^) was chosen per site. At Naranjal, all plants were of the Castillo variety, which is the most common in Colombia. This variety is composed of 39 breeding lines with similar agronomic attributes selected from F5 and F6 generations after crossing the Caturra variety and the Timor Hybrid (Alvarado et al., 2005). At Paraguaicito, the plantation consisted of representative breeding lines of the same variety but identified by rows, which allowed choosing favored plants from the specific lines CU 1855 and CX 2710.

Based on the recommendations of the fertilization plan for coffee crops during the vegetative stage, 2 months after transplanting each coffee seedling received an application of 10 grams of urea with an enrichment of 2.4 atom % ^15^N, equivalent to a single rate of 100 kg.ha^−1^. Coffee crops at this stage are fertilized with 10–35 g of urea per plant at 2, 6, 10, 14, and 18 months, and rates are given by plant (g.plant^−1^) in contrast to the recommendation for the productive stage which is given by area (kg.ha^−1^). Knowing that N fertilization is always required by the coffee crop and that N is partially lost when urea is surface applied, two fertilizer application methods were evaluated per site: (1) *the surface applied method* consisted of leaving the fertilizer uncovered in a small band 25 cm long and 5 cm deep at 15 cm from the stem, similar to current practice, and (2) *the incorporated method*, in which fertilizer was applied in a similar band (25 cm long and 5 cm deep) but covered manually with soil, as an alternative to reduce N volatilization and increase use efficiency. A non-fertilized control was not included due to the fact that by using ^15^N, N uptake from fertilizer is quantified directly from the isotopic enrichment of the plants.

Sixteen plants in Naranjal and 20 in Paraguaicito with homogeneous growth were chosen from two contiguous rows, and a completely randomized design with eight and ten replicates per application method was used at Naranjal and Paraguaicito, respectively.

For all plants (replicates), one of the first fully expanded leaves from the top was harvested at 10, 20, 60 days following fertilization. The experiment was terminated at this time (4 months following fertilization), which corresponds to the time when a second N application is typically applied to coffee plantations. Total roots, sampled by carefully removing the whole plant from the field using a watering can, as well as stems, branches and leaves for every plant were sampled separately, weighed fresh and dried at 60°C. The dry mass of all components was recorded and root to shoot ratio (R:S) was calculated. All components were dried, milled, and sent to the Stable Isotope Facility at the University of California, Davis, where the contents of N, ^15^N and ^13^C were determined by dry combustion-gas chromatography-isotope ratio mass spectrometry. The following calculations were performed for each plant component (root, stem, branch and leaf).

The percent of plant N derived from fertilizer (Ndff) was calculated as:

$$\%\text{Ndff}=\left(\begin{array}{l} \frac{\text{atom}\%{\;}^{15}\text{N excess in plant sample}}{\text{atom}\%{\;}^{15}\text{N excess in fertilizer}}\\ \; \end{array}\right)\text{ x }100$$

where atom% ^15^N excess is the measured ^15^N content of the plant sample minus the background ^15^N content before fertilization.

Nitrogen use efficiency (NUE), also referred to as recovery of fertilizer N in plants, was calculated as:

$$\text{NUE}=\left[\frac{\text{mass N in plant component x}\left(\frac{\%\text{Ndff}}{100}\right)}{\text{mass fertilizer N applied}}\right]$$

The macronutrient content (concentration in %) of each component was determined in additional subsamples by digestion and atomic absorption spectrometry at the Soils Lab of Cenicafe following methodologies described by Carrillo et al. (1994). Total macronutrient uptake (accumulation in g) was calculated by multiplying nutrient concentration and dry mass of each component.

All data were analyzed together and by each plant component in order to test simple effects of site and application method, as well as the interaction (site^*^method) effect using general linear models with the SAS software package (SAS, 2004). To test normality of residuals and homogeneity of variances, the Kolmogorov-Smirnov and Levene's tests were used for each parameter; means and standard errors of all parameters were calculated for each site and application method, and the effect of each method was estimated using a two-way ANOVA with a level of significance of 5%. A Tukey test was used to compare Ndff and ^13^C means through time and LSD tests were used to compare means of all other variables between application methods and between sites.

## Results

For all the variables, part of the statistical analyses is presented in Tables 2–4. Ndff through time was more increased by the surface application method in Naranjal, and both sites registered the highest Ndff and the lowest ^13^C values 60 days after planting (DAF). Table 3 shows that most of variables measured in the four plant components (root, stem, branch, and leaf) were only affected by the site source of variation and that no effects of the application method, except on Ndff, were registered. Final Ndff exhibited main effects from site and application methods, and NUE was different only among components. However, NUE, dry weight and macronutrient uptake (accumulation-g) of both aboveground and whole plant biomass were not affected by any source of variation (Tables 3, 4).

**Table 2:** **Pr > ***F***-values from the statistical output (Anova, 5%) for the Ndff and leaf ^**13**^C variables through time (DAF: Days after planting)**.

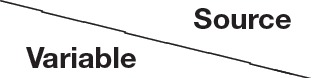	**Site**	**Method**	Site^*^Method	**DAF**	Site^*^DAF	Method^*^DAF	Site^*^Method^*^DAF
Ndff	**<0.0001**	**0.0066**	0.2942	**<0.0001**	0.8189	0.6878	0.7683
Leaf ^13^C	**0.0007**	0.1285	0.6971	**<0.0001**	0.9275	0.9872	0.6061

*Bold values indicate significant effects*.

**Table 3:** **Pr > ***F***-values from the statistical output (Anova, 5%) for all variables at day 120 by each plant component**.

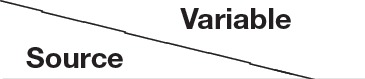	**Ndff 120d**	**NUE**	**Dry weight**	**N (%)**	**P (%)**	**K (%)**	**Ca (%)**	**Mg (%)**
**ROOT**								
Site	**0.0011**	0.4547	**0.0002**	**0.0082**	**0.0029**	**<0.0001**	**0.0016**	0.0173
Method	**0.0159**	0.3801	0.3789	0.3046	0.6160	0.5982	0.5962	0.4173
Site^*^Method	0.4281	0.4806	0.5102	0.7915	0.3540	0.7777	0.9244	0.6240
**STEM**								
Site	**0.0087**	**<0.0001**	0.1351	**<0.0001**	0.2054	**<0.0001**	**0.0016**	**<0.0001**
Method	**0.0206**	0.1292	0.7287	0.3924	0.8945	0.6392	0.4038	0.4810
Site^*^Method	0.3688	0.8930	0.1748	0.2653	0.5664	0.4598	0.6480	0.8596
**BRANCH**								
Site	0.2427	0.0261	0.0574	**<0.0001**	**0.0013**	**0.0002**	0.6730	**<0.0001**
Method	0.0321	0.4968	0.3685	0.7557	0.4057	0.2197	0.3039	0.7117
Site^*^Method	0.7878	0.3768	0.2717	0.0766	0.6619	0.2764	0.1942	0.9109
**LEAF**								
Site	0.3941	0.7396	0.5043	**<0.0001**	**<0.0001**	0.9461	0.5517	**<0.0001**
Method	0.0698	0.8992	0.2695	0.2595	0.6544	0.0517	0.5990	0.9267
Site^*^Method	0.7608	0.2243	0.2178	0.9215	0.9108	0.2513	0.5853	0.4101
**ABOVEGROUND BIOMASS**								
Site	0.1220	0.1734	0.7132	**<0.0001**	0.6302	**0.0169**	0.1477	**<0.0001**
Method	0.0338	0.8305	0.3427	0.4698	0.5283	0.0929	0.2084	0.9656
Site^*^Method	0.7997	0.2732	0.1739	0.2807	0.7496	0.2317	0.1680	0.5576

*Bold values indicate significant effects*.

**Table 4:** **Pr > ***F***-values from the statistical output (Anova, 5%) for all variables at day 120 for the whole plant**.

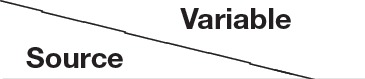	**Ndff**	**NUE**	**Dry weight**	**R:S ratio**	**N uptake**	**P uptake**	**K uptake**	**Ca uptake**	**Mg uptake**
**WHOLE PLANT**									
Site	0.0510	0.2741	0.1520	**0.0004**	0.5652	0.0325	0.1830	0.0678	0.9578
Method	**0.0273**	0.7383	0.3195	0.8712	0.3446	0.3118	0.7101	0.3026	0.1361
Site^*^Method	0.7113	0.2767	0.2086	0.3234	0.1210	0.1300	0.2590	0.3784	0.2961

*Bold values indicate significant effects*.

### N use efficiency (NUE)

**Figure 2:**
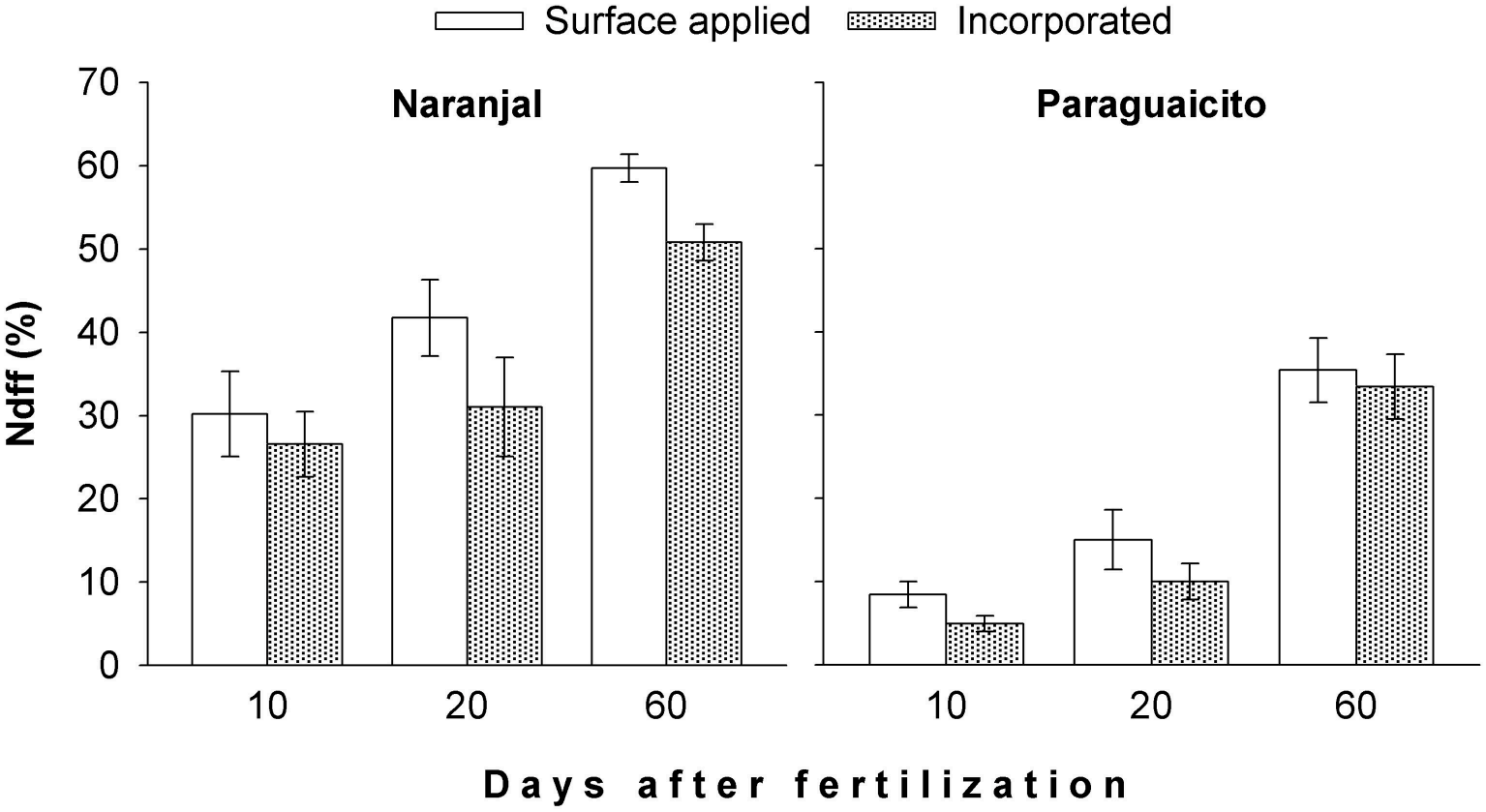
**Effect of fertilizer application method on percent of coffee plant N derived from fertilizer (Ndff) through time at two contrasting sites, as indicated by the first fully expanded leaves from the top**. Bars show standard errors.

The percent of N derived from fertilizer (Ndff) in the more active top leaves was consistently greater at Naranjal compared to Paraguaicito during the entire evaluation period, and a significant effect (*p* = 0.0066) of the urea application method on this variable at day 60 was registered at Naranjal (Figure 2). Ndff was higher for the surface method and increased through time until day 60 after urea application when the highest Ndff was observed.

**Figure 3:**
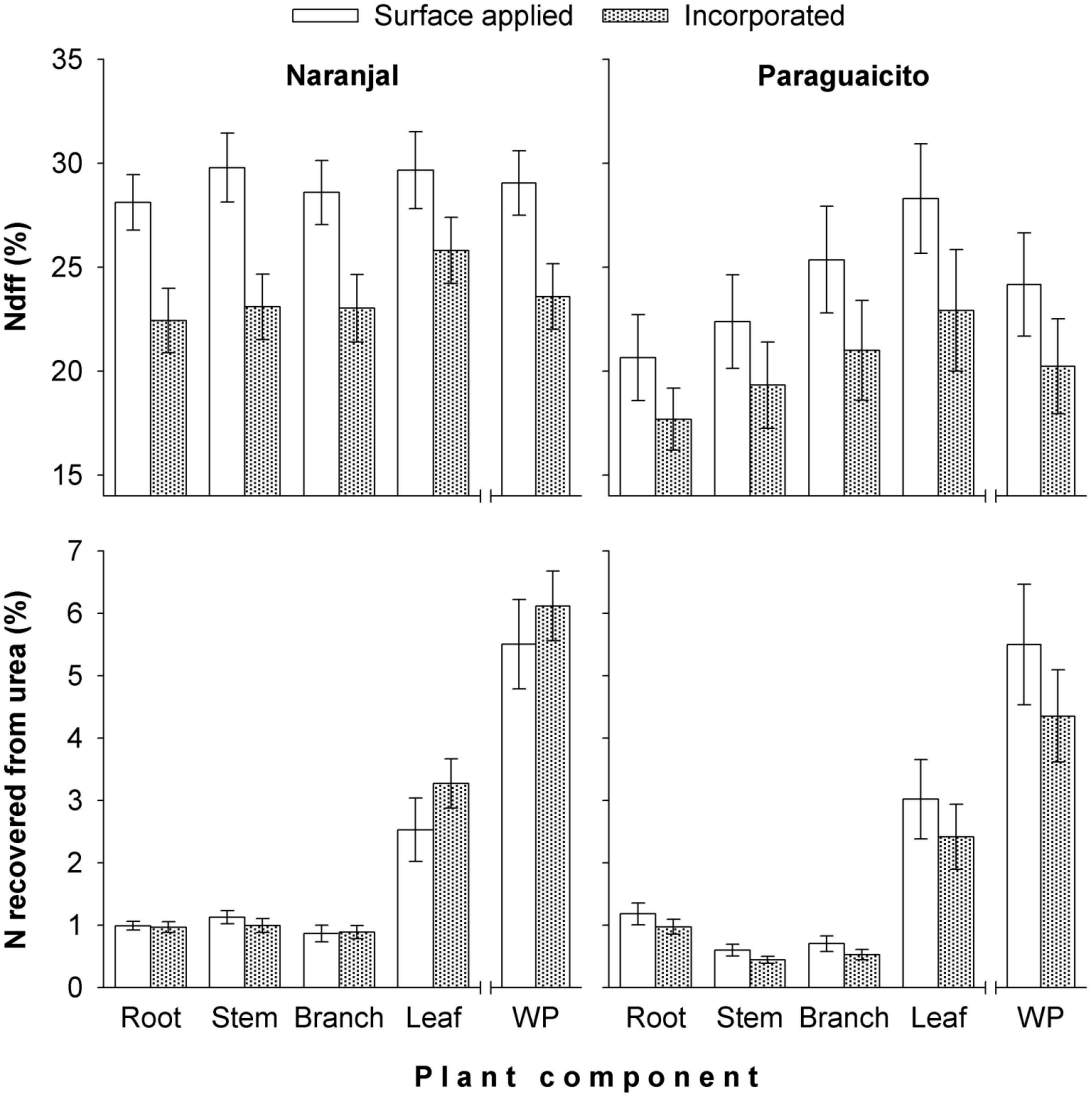
**Effect of fertilizer application method on percent of coffee plant N derived from fertilizer (Ndff) and recovery of N from fertilizer (NUE) after 120 days in different components and the whole plant (WP) grown at two contrasting sites**. For Ndff, WP corresponds to the weighted average of all components, while for N recovery, WP corresponds to the sum of all components. Bars show standard errors.

The final Ndff and recovery of N measured at 120 days from fertilization is shown in Figure 3. Coffee plants at Naranjal showed a higher percent of Ndff in roots (*p* = 0.0011) and stems (0.0087) than plants in Paraguaicito, and surface application also resulted in significantly higher Ndff measured in roots (*p* = 0.0159), stems (*p* = 0.0206) as well as in the whole plants (*p* = 0.0273). However, no significant effects of both site and application method were registered on Ndff values of leaves and branches. The proportion of urea N recovered by whole plants (NUE) was not affected by the application method, but a significant site effect was registered for the stems (*p* < 0.0001), with a higher recovery found at Naranjal than Paraguaicito.

For both sites, a low NUE was registered during the initial phase of the vegetative stage since coffee plants took up only an average of 5% of the urea-N application; this amount of fertilizer-derived N constituted 24–29% (Naranjal) and 20–25% (Paraguaicito) of the total N in the plant at the end of the experimental period (Figure 3). Based on these results, we reject our hypothesis since NUE was similar between sites and also between methods, despite their contrasting crop yield response to N fertilization.

### Biomass accumulation and allocation

**Figure 4:**
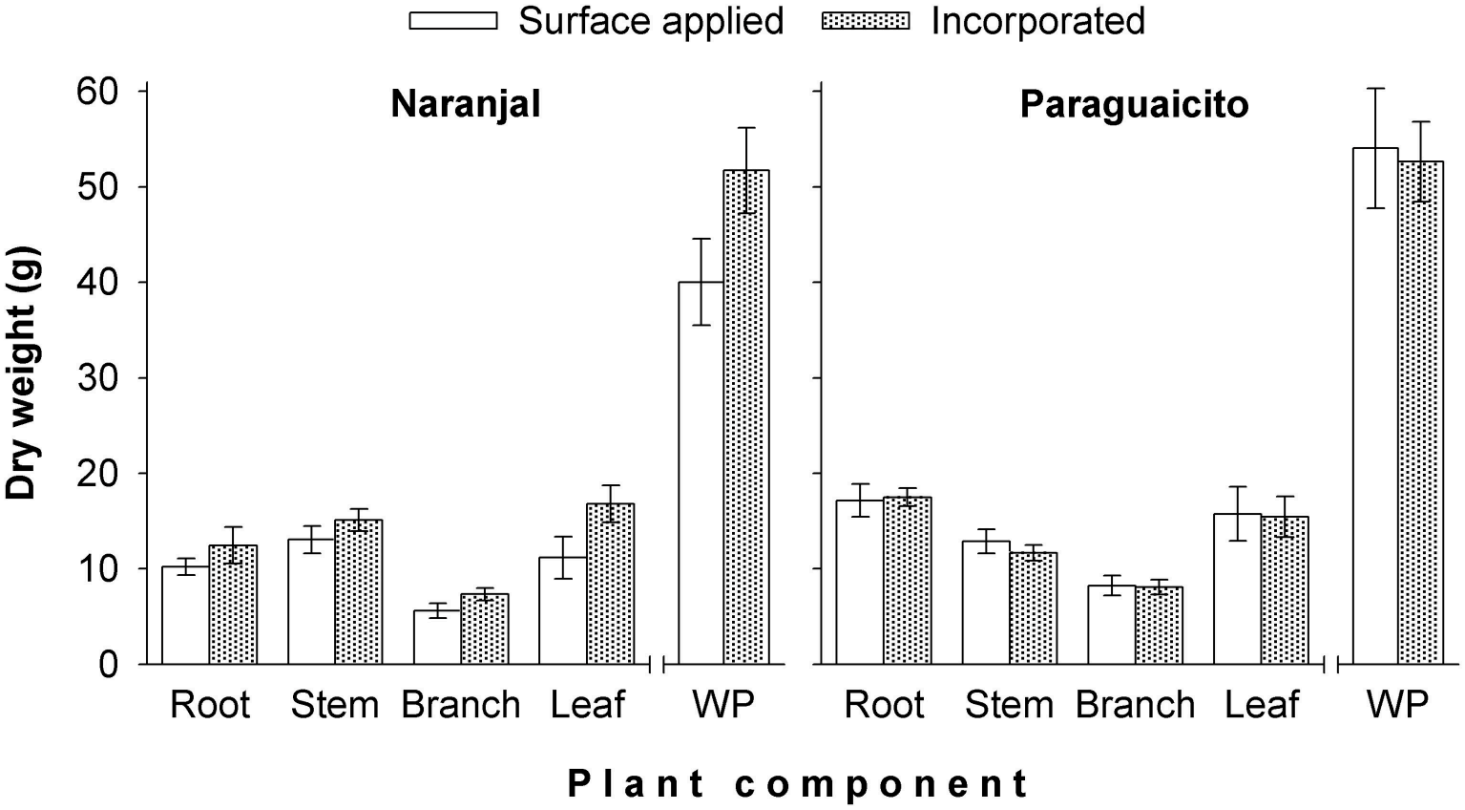
**Effect of fertilizer application method on biomass allocation and total mass (WP) in coffee plants grown at two contrasting sites**. Bars show standard errors.

**Figure 5:**
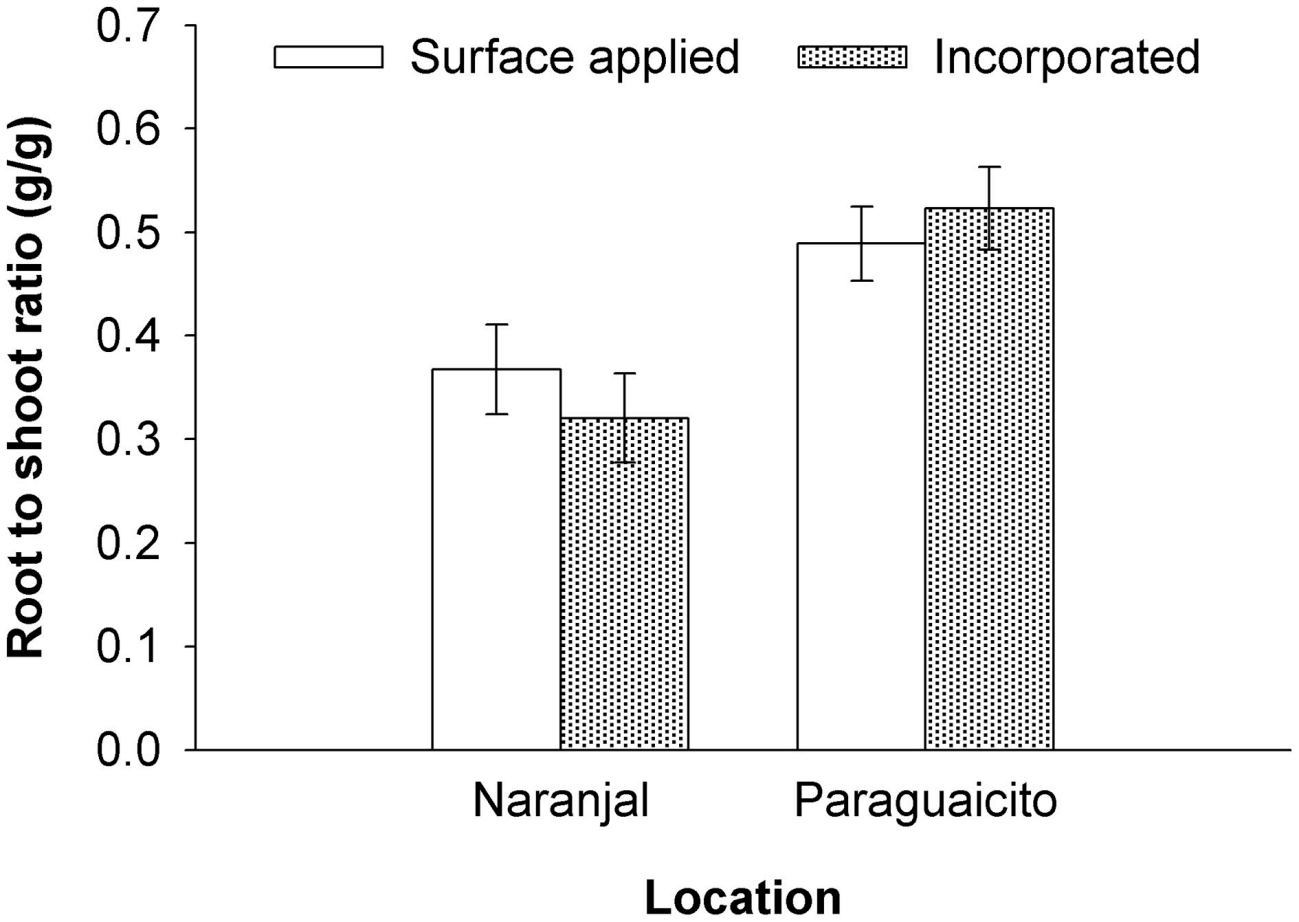
**Root to shoot ratio in coffee plants fertilized by two methods at two contrasting locations**. Bars show standard errors.

As presented in Table 3 and Figure 4, a significant site effect was observed for dry weight only for the root component and mass allocation was different between sites. Plants allocated more biomass to shoots at Naranjal but registered a higher allocation to root systems at Paraguaicito. Related to the application method effect, no significant effect was registered on the dry weight of the four components or even the whole plant. In general, plants grown at Naranjal exhibited a lower root mass as well as a significantly lower root to shoot ratio (Table 4) compared to plants grown at Paraguaicito, but no effect of application method was registered on this ratio at both locations (Figure 5).

### Effect of N application on macronutrient uptake

Macronutrient concentrations in the different plant components were affected only by site, and no effects of the application method were registered (Table 3). As indicated in Table 5, higher concentrations of N in roots, stems, branches, leaves, and therefore of aboveground biomass, were registered at Naranjal while higher P concentrations were observed in roots and leaves at Paraguaicito. With the exception of roots, concentrations of K, Ca, and Mg in stems, branches and leaves of coffee seedlings exhibited an opposite response to soil fertility. At Naranjal with lower K, Ca, and Mg soil concentrations, higher concentrations of K, Ca, and Mg in stems, K and Mg in branches and Mg in leaves, as well as higher K and Mg in aboveground biomass were registered, whereas higher K and Ca concentrations were registered in roots at Paraguaicito where the soil also had a higher base content.

**Table 5:** **Macronutrient uptake in terms of concentration (%) and total accumulation (g) in coffee plant components, aboveground biomass (AG) and whole plants (WP) after surface-applied and incorporated N fertilizer for both experimental sites**.

**Site**	**Plant Comp**.	**N**		**P**		**K**		**Ca**		**Mg**	
		**%**	**g**	**%**	**g**	**%**	**g**	**%**	**g**	**%**	**g**
Naranjal	Root	1.53a	0.17	0.08b	0.01b	0.89b	0.10b	0.54b	0.06b	0.27	0.03b
	Stem	1.14a	0.16a	0.09	0.01a	1.01a	0.14a	0.33a	0.05	0.12a	0.02a
	Branch	2.12a	0.14a	0.18a	0.01	2.42a	0.16	0.50	0.03	0.26a	0.02
	Leaf	3.31a	0.46	0.15b	0.02	2.18	0.30	1.10	0.15	0.48a	0.07
	AG	2.19a	0.76	0.14	0.05	1.87a	0.60	0.65	0.23	0.28a	0.10
	WP	2.02	0.93	0.13	0.05	1.62	0.70	0.62	0.29	0.28	0.13
Paraguaicito	Root	1.36b	0.24	0.10a	0.02a	1.34a	0.23a	0.75a	0.13a	0.34	0.06a
	Stem	0.75b	0.09b	0.08	0.01b	0.82b	0.10b	0.27b	0.03	0.06b	0.01b
	Branch	1.25b	0.10b	0.14b	0.01	2.01b	0.16	0.48	0.04	0.15b	0.01
	Leaf	2.83b	0.44	0.20b	0.03	2.17	0.32	1.07	0.17	0.30b	0.05
	AG	1.61b	0.63	0.14	0.05	1.67b	0.59	0.61	0.24	0.17b	0.07
	WP	1.55	0.87	0.13	0.07	1.58	0.81	0.64	0.37	0.21	0.13

*For nutrient concentration, AG and WP correspond to the weighted average of their respective components, while for g, AG and WP corresponds to the sum of their components*.

*Values with different letters indicate statistical differences among sites per each plant component (LSD test, p < 0.05)*.

In terms of total nutrient accumulation (g) related to dry biomass, data followed a similar trend as the concentration values registering less significant effects between sites and no effects of the application method. In general, as shown in Table 5, coffee plants grown at Naranjal accumulated higher amounts of N (*p* < 0.0001), P (0.0142), K (*p* < 0.0007) and Mg (*p* < 0.0001) in the stems as well as more N (0.0143) in the branches, whereas plants grown at Paraguaicito accumulated more P (*p* < 0.0001), K (*p* < 0.0001) Ca (*p* < 0.0001) and Mg (*p* < 0.0001) in roots. Conversely, total accumulation of macronutrients in the aboveground biomass and the whole plants were not affected by site or application method (Tables 3, 4).

### ^13^C content

**Figure 6:**
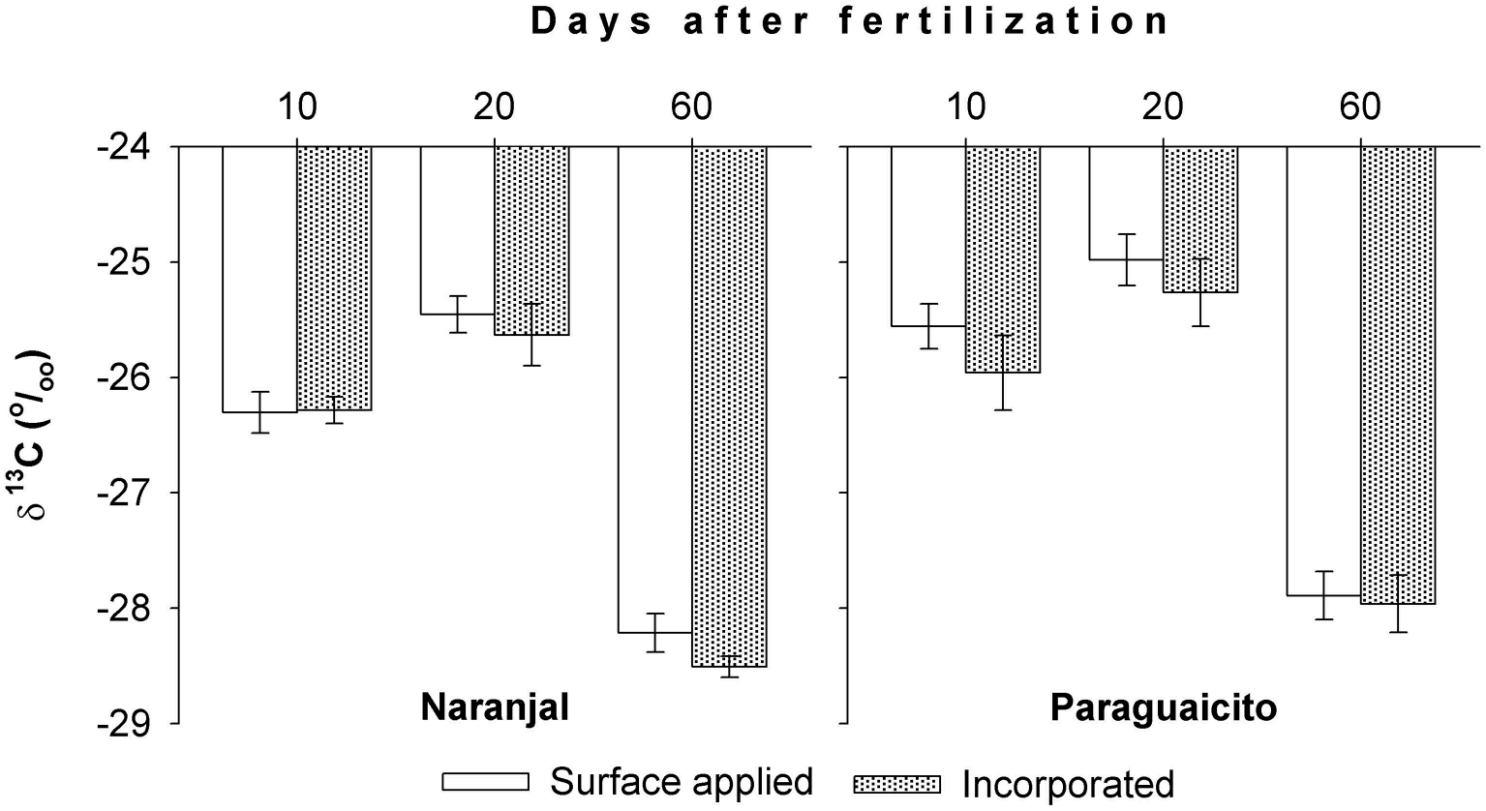
**Leaf ^**13**^C content through time in coffee plants fertilized by two methods at two contrasting locations, as indicated by the first fully expanded leaves from the top**. Bars show standard errors.

Values for δ^13^C, an integrated measure of physiological factors influencing water uptake during the study period, of the first fully expanded leaves from the top are presented in Figure 6. The more positive values observed at 20 days reflect the decrease in precipitation at both sites, while the more negative values indicate greater water availability during the subsequent period, up to 60 days, where the lowest leaf ^13^C values were registered for both sites. This variable exhibited also a site effect, with higher values in Paraguaicito where less rainfall and resulting lower soil moisture were registered, but no differences as a consequence of the fertilizer application techniques.

## Discussion

### N use efficiency

The amount of N derived from fertilizer (Ndff) through time was associated with soil moisture in both locations as well as plant growth response to the environmental conditions. Ndff after 2 months followed similar trends as rainfall, which likely increased soil moisture, since higher values were registered at Naranjal (Figure 2), where total precipitation was higher. Such high precipitation registered after application (Figure 1) likely distributed the surface-applied urea along the soil surface more widely than in the incorporated application method, where the fertilizer remained more concentrated in a smaller area less accessible to plant roots. This trend was also related to the variation in the ^13^C content through time (Figure 6), which reflects specific physiological changes and C assimilation in leaves resulting from changes in water availability at the root zone (Condon et al., 2004). Lopez et al. (2002) state that under adequate soil moisture, mineralization of native N is enhanced by fertilization, which affects fertilizer use efficiency. A possible effect of N fertilizer on mineralization rate is increased N uptake from the native pool, described by Jenkinson et al. (1985) as the priming effect or Apparent Added Nitrogen Interaction, which is caused by the diverse soil processes affecting the N cycle, including plant uptake, and is likely why NUE values estimated using the isotopic method are often lower than those estimated indirectly. This effect also becomes even more important for Colombian soils, where organic matter content can reach up to 30% and is the main edaphic factor associated with coffee response to N fertilizers, as described by Sadeghian (2008).

It is important to better understand the effect of edaphic and environmental conditions on nutrient uptake and NUE, since coffee is a perennial crop grown in tropical regions with seasonal variation in terms of water availability, and since fertilization is recommended twice a year. According to Martinez-Alcantara et al. (2012), late application of nutrients in summer instead of spring months increased NUE in 5-year-old orange trees because fertilizer-derived N increased in the tree storage organs and remained available the following spring when growth resumed. In light of such conditions, fertilization at the end of the rainy season when lighter rains occur, rather than at the beginning or during the heavy rainy periods as currently practiced, could be considered and remains to be validated as a strategy to increase longer-term NUE in coffee.

Our study shows a low recovery of fertilizer N (NUE) at both locations in each of the plant components after 6 months, and consequently in the total biomass. Leaves accumulated about 50% of the total N recovered in the whole plants. Similarly, Suarez (1996) report that only 8% of fertilizer N was recovered by coffee plants, of which 59.7% was found in leaves followed by 21.7% in branches, 9.9% in stems, and 8.8% in roots. Most (approximately 95%) of the fertilizer N remained in the soil or was lost by surface runoff or leaching through the profile, considering the high levels of precipitation during the evaluation period (Figure 1).

Although no measurements were taken to estimate residual fertilizer N in the soil, diverse studies even in annual crops show that this component is low and that plants do not rely totally on it. For example, Kumar and Goh (2002) give ^15^N recoveries in wheat and ryegrass of 52% and 41%, losses attributed to leaching and denitrification of 12 and 24%, and recovery of residual fertilizer by a subsequent wheat crop of only 1–5%. According to Fritschi et al. (2005), N recovery in cotton after three seasons was lower than 5%. Under mid-hill conditions of Nepal, Pilbeam et al. (2002) did not register any effect of rate or form of N inputs in maize but observed a low N recovery (<25%) with only 3% recovered by a subsequent millet crop, also indicating little contribution of fertilization to subsequent crops.

Based on the fertilization plan already established for coffee at the vegetative stage, a second urea application is typically prescribed 6 months after transplanting (Sadeghian, 2008). This means that at the end of the current study, the plants had reached the point where a higher dose of N (15 grams) is typically applied, and although N recovery from urea might be higher than for the first N input due to the plant size, the cumulative effect of N fertilizer is expected to be similar, with high losses to the environment. More measurements at the field level and over longer periods of time are required to more accurately account for loss and therefore better estimate overall fertilizer use efficiency.

### Biomass accumulation and allocation

In a previous study under greenhouse conditions (Salamanca-Jimenez, 2015), coffee seedling growth was more affected by N dose than by soil moisture. Root to shoot ratio decreased significantly with increasing N at all soil water levels but was higher for all N levels under drier soil conditions. A similar effect was registered in the current study for the root to shoot ratio, since higher values were registered in Paraguaicito, where a combined effect of lower soil moisture and lower nutrient availability due to the lower precipitation, lighter texture and lower soil fertility drove plants at this location to relocate more biomass to roots. In contrast, at Naranjal, plants produced more shoot biomass in response to the higher water and nutrient availability, especially N and Mg which registered higher contents in stems, branches and leaves for this location.

Furthermore, the significant effect registered on branch and leaf dry weight at Naranjal is likely associated with a reduction of losses when fertilizer was incorporated. According to Mahler and Hamid (1994), as moisture increases the rate of hydrolysis of urea also increases and N losses can reach up to 80% if urea is applied on the soil surface, while Kissel (1988) state that with incorporation of urea at a sufficient depth NH_3_ losses may become negligible.

Losses in our study were not measured but a study under similar soil and climate conditions by Leal (2007) reported N losses of up to 40% of the applied N and a reduction of losses when fertilizer was covered with litter. Depending on rainfall amount and distribution patterns, it is probable that if losses are reduced by incorporating lower doses of fertilizer, plants could still obtain adequate N and reach a similar biomass during the initial growth stages, which would not compromise yield but would reduce the negative environmental impacts of excess urea application.

### Macronutrient uptake

Although the fertilizer doses were similar for both locations, plants at Naranjal registered higher concentrations of N in all plant components (roots, stems, branches and leaves). Nevertheless, the lack of effect of the urea application method on macronutrient contents of coffee plants is related primarily to the lack of effect on the total N uptake in the whole plant, since synergistic as well antagonistic effects of fertilization usually occur during nutrient uptake at the root level (White, 2012). For instance, bentgrass plants fertilized with ${\text{NO}}_{3}^{-}$ or ${\text{NH}}_{4}^{+}$ or both exhibited variable concentrations of macronutrients. Compared to ${\text{NH}}_{4}^{+}$-treated plants, ${\text{NO}}_{3}^{-}$-treated plants accumulated higher concentrations of K, Ca, Mg in shoots, higher concentrations of P, K, Ca, Mg in roots, and higher concentrations of P, Ca, Mg in the verdure tissue (McCrimmon et al., 1992).

In spite of these relationships, nutrient content in plant components should not be considered independently. Other factors such as mobilization, variety, age, or shade level are also involved; Thornton et al. (1999) state that N content in shoots is affected by shoot water content and declines due to its mobilization from the leaves to new tissues as the growing season progresses. McCrimmon (2002) report a wide variation of macronutrient contents among bermudagrass cultivars, and according to Jacques et al. (2007), such concentrations may be also affected by tissue age and shade patterns.

In our study the variation in macronutrient concentrations between sites was related to the differences in soil fertility and climatic conditions and their resulting effect on root growth. Higher N concentration in all the plant components, and therefore higher N status of the coffee plants, as well as higher N, K, and Mg concentrations of aboveground biomass, along with the lower root to shoot ratio in Naranjal compared to Paraguaicito, likely resulted from higher N availability associated with higher soil N mineralization; this in turn resulted from higher soil organic matter content and rainfall at Naranjal (Figure 1). Plant nutritional status reflected the combined effect of all these factors, and a more beneficial impact of the intrinsic soil fertility (OM, CEC) on nutrient uptake than the fertilizer incorporation method.

## Conclusions

In coffee plants at the initial phase of vegetative growth, our results showed the combined effect of site and method on Ndff in roots and stems and only a site effect on root mass, root to shoot ratio, and macronutrient content. A higher percent of Ndff in roots and stems was registered at Naranjal by the surface application method. Additionally, the higher root mass, root/shoot ratio, P, K, and Ca concentration of roots and P of leaves at Paraguaicito, and the higher N, K, and Mg concentration in the aboveground biomass at Naranjal were associated with a combined effect of lower soil moisture and lower fertility on coffee plant growth and nutrient allocation.

Nevertheless, the lack of effect on recovered N does not necessarily indicate that fertilizer incorporation should be avoided and surface application should be continued. NUE values for both methods were low, and other factors, mainly related to immobilization and/or losses, determine the ultimate viability of a fertilization approach.

Four months following fertilization, approximately 25% of the N in the top leaves was derived from this application, but only a small fraction of the total amount of N applied was observed in the whole plants (an average NUE of 5%). Thus, immediate efforts toward increasing NUE during initial establishment and even the entire vegetative crop stage must focus on evaluation of multiple levels of fertilizer combined with different application methods. Future trials may include typical fertilizer rates (such as those used in the present study) as well as proportional lower rates, in order to establish threshold values that consider short-term plant uptake and longer-term soil supply.

N fertilization is always recommended in coffee but its effect is likely indirect, in that it stimulates mineralization and uptake of native soil N. Further studies of this “priming” effect, with even lower doses of N, along with immobilization and retention of residual fertilizer N, will contribute to a better understanding of N cycling under Colombian conditions, and will lead to more effective fertilizer strategies for matching crop needs and reducing environmental impacts.

## Author contributions

AS, TD, and WH conceived the experiment; AS and TD performed the experiment; WH provided materials; AS wrote the first version of the manuscript; all authors contributed to the final manuscript.

### Conflict of interest statement

The authors declare that the research was conducted in the absence of any commercial or financial relationships that could be construed as a potential conflict of interest.
